# A primer to ‘bio-objects’: new challenges at the interface of science, technology and society

**DOI:** 10.1007/s11693-013-9104-8

**Published:** 2013-02-26

**Authors:** Peter Dabrock, Matthias Braun, Jens Ried, Uwe Sonnewald

**Affiliations:** Erlangen, Germany

**Keywords:** Synthetic biology, Ethics, Bio-objetcs, Societal questions, Moratorium

## Abstract

Biotechnological and life science innovations do not only lead to immense progress in diverse fields of natural science and technical research and thereby drive economic development, they also fundamentally affect the relationship between nature, technology and society. Taken this seriously, the ethical and societal assessment of emerging biotechnologies as for example synthetic biology is challenged not only to constrain on questions of biosafety and biosecurity but also to face the societal questions within the different fields as an interface problem of science and society. In order to map this vague and stirring field, we propose the concept of bio-objects to explore the reciprocal interaction at the interface of science and society serious as well to have the opportunity to detect possible junctions of societal discontent and unease before their appearance.

Synthetic biology seemed to be on the safe side. All stakeholders’ statements—the European as well as the US American—expose that “[…] no new regulations are needed, but […] officials should be vigilant in case bigger risks arise in the future” (Presidential Commission for the study of Bioethical Issues 2010). Even more, the statement of the German Research Foundation (DFG), Leopoldina and Acatech, concludes that “with respect to biological safety […] and the risk of misuse […] the existing legislation in Germany is sufficient for the present state of research” (Deutsche Forschungsgemeinschaft et al. 2009). Simultaneously the Danish Council of Ethics as well as The Danish Board of Technology assess that “synthetic biology involves only limited risks in its present stage, and there is currently no need for new legislation for this particular area” (The Danish Council of Ethics, The Danish Board of Technology 2011). Of course it is necessary to carefully survey the ongoing development due to the possible ethical as well as societal risks, but nevertheless there are no general objections concerning ongoing research in synthetic biology. Common to this scientific evaluation of synthetic biology, all scientific statements tag the public at large and its attitude towards synthetic biology in particular as a crucial point. In sum, the story seems to require awareness on a societal level, but due to the scientific assessment nevertheless seems to be unproblematic overall.

Currently, the situation is changing: the supposedly neutral or even positive societal perspective on and evaluation of synthetic biology is at risk. 111 civil society organizations (CSO) call for a moratorium on synthetic biology: “The Precautionary Principle must be applied to synthetic biology because the risks of the technology are inherently unpredictable with potentially far-reaching and irreversible impacts” (Friends of the Earth U.S., International Center for Technology Assessment, ETC Group 2012). Due to such an argumentation the CSOs call for a moratorium “on the release and commercial use of synthetic organisms and their products” (Friends of the Earth U.S., International Center for Technology Assessment, ETC Group 2012). From a scientific perspective, the story still remains clear: Nothing has changed! The researchers are still working with microbes and bacteria, no accident has happened up to now and biosafety as well as biosecurity are still ensured. Nevertheless, synthetic biology might no longer be restrained in the lab, it seeks to carve out and begins to ‘act’ in between science and society. The different scientific, societal and economic actors are put in a flurry: Will synthetic biology suffer the same fate as the so called ‘green biotechnology’? How can this vague and stirring field be mapped?

It perfectly fits the problem that heretofore there was no detailed knowledge about which values and issues in regard to synthetic biology can be found in the public and which of those were relevant to what extent (Pauwels 2009). At the same time, the few studies that decidedly deal with values and opinions state that the general public is largely ignorant and unaware of what synthetic biology is. Thus, the results of the latest Eurobarometer make clear that 83 % of the Europeans have not noticed the topic of synthetic biology yet and do not have any substantial ideas about it (Gaskell et al. 2010). Studies from the USA show that there has been some change in the perception of synthetic biology: in 2010, 26 % of the interviewed people had already heard about synthetic biology, whereas in 2008 only 9 % had done so (Hart Research Associates 2010). With regard to the most important aspects addressing the future development of synthetic biology 33 % believe that the risks and benefits will be about equal (33 %), while 19 % think the benefits will outweigh the risks and 16 % state the risks will exceed the benefits (Hart Research Associates 2010). Such investigations that show a lack of knowledge about synthetic biology present both opportunities and risks. Opportunities arise due to the fact that the lack of knowledge offers a possibility to inform society seriously and risks arise in respect to the societal inability to detect and uncover scandalization strategies by the sciences, civil society organizations or the media.

Apart from these observations of a broad lack of knowledge, there are descriptions of discontent that can be detected not only in the terminology of the media but also in the remarks of some scientists (Biotechnology and Biological Sciences Research Council, Engineering and Physical Sciences Research Council 2010). The described discontent of the public accumulates to a fear of unknown safety risks for one thing and to a general concern or unspecified expectations concerning the implicit moral consequences of synthetic biology itself for another thing (Yearley 2009). Accordingly, Bedau et al. (2008) summarize: “Thus, the prospect of artificial cells can be expected to generate widespread unease, distrust and even hostility in the public at large”. This discontent is to a high extent fueled by the fact that the promised applications of synthetic biology are currently highly speculative visions (Presidential Commission for the study of Bioethical Issues 2010).

In the current scientific debates, the prominent approach dealing with the described discontent in the public is the one that states that the public’s perception of synthetic biology is generally linked to the extent of focus on advantages and expected profits by the media and by individual valuations: “The balance between potential risks and benefits seems to be the basis for public confidence in synthetic biology” (European Group on Ethics in Science and New Technologies to the European Commission 2009). If this appraisal is put straight, the descriptions of the rising discontent is endangered to be nothing more than just marginal phenomena of an exaggerated vision that in retrospect would be described as background noise due to fulfilled expectations or falsified visions. Taken this assumption seriously our exposed problem still remains: How to explain the emerging discontent and intensive reaction of society in general and the 111 CSOs in particular?

The considerations so far emphasize that the societal discontent has to be taken serious in order to map the interface of science and society. As shown above, societal discontent fulfills the function of an indicator for trans- and reconfiguration of traditional and taken-for-granted-distinctions such as ‘living’ vs. ‘non-living’, ‘artificial’ vs. ‘natural’ or ‘subject’ vs. ‘object’. These distinctions have hitherto been deemed as irrefutably valid on the level of processing on the one hand and on the level of expressing on the other hand. Here it is necessary to distinguish within the societal discontent: On the one hand there is a kind of more conscious—and therefore superficial—concern which is mainly related to risk perceptions (or imaginations) regarding the new technical procedures of synthetic biology. On the other hand there is a kind of deeper and subconscious unease that can be considered as an expression of taken-for-granted-assumptions. Regarding the first differentiation (the level of procedure and biotechnology), concerns concentrate on the new technical developments, and thus it focuses on the continuous and discontinuous classification of these technical procedures in comparison to earlier procedures (Kelle 2009). Such a comparison—coinciding with a strong reliance on a precautionary principle—is always in danger of regarding a new development extremely sceptically in a yet early stage. So as to deal with this first differentiation of discontent, a focus on classical questions of biosafety and biosecurity is necessary even if it is not sufficient to discuss the function of discontent thoroughly.

In order to grasp the full impact of the above mentioned discontent it is necessary to consider the second differentiation inscribed in the debates on biosafety and biosecurity. It takes the form of a “deep grammar” (Wittgenstein 1922) which neither scientific nor public debates have so far explicitly regarded and which has still been considered only marginally by ethical research. Such a “deep grammar” can be characterized as unease in regard to the disruption of previous taken-for-granted distinctions between ‘living’ versus ‘non-living’ matter, ‘artificial’ versus ‘natural’ as well as ‘organic’ versus ‘non-organic’ (Ried et al. 2011). Such an unease is triggered off by certain branches of synthetic biology—especially research with protocells—which could be able to create or claim the creation of epistemological and ontological entities that cross out previous distinctions and dichotomies. This fact evokes unease about which categories and terminologies to use when describing these transformations adequately—both in terms of science and in terms of life world’s language.

Taken seriously, both societal concern and unease as obligatory parts of the societal discontent are not just another additional ‘nice-to-have’ on the ‘ethical playlist’—nevertheless to ensure the own crack of the (funding) whip—but they rather draw concrete consequences for the assessment of emerging biotechnologies as it can be elucidated in the field of ‘plant-based synthetic biology’. Plant-based synthetic biology aims at creating novel or optimal production systems for bioactive metabolites and polypeptides. Considering an energy-efficient and CO_2_-efficient production, approaches utilizing natural photosynthesis are best suited (Biemelt et al. 2003). These light-driven processes can also be used for the sustainable production of anti-microbial peptides. Anti-microbial peptides have a great potential as substitutes for conventional antibiotics (Planson et al. 2011). There are two prerequisites for developing next-generation antibiotics: identification or de novo synthesis of bioactive polypeptides and the development of optimal bioreactors for their production. Novel anti-microbial peptides are either found in nature or designed by in silico simulation studies (biased), molecular evolution (unbiased), or by the construction of chimeric peptides (combinatorial). Following the identification or the design of novel bioactive polypeptides, synthetic genes promise to allow efficient expression in target cells, which are created and used for the generation of transgenic cells. Such target cells may be higher plant-derived or algae-derived and by processing and molecular engineering, optimal product levels and security can be achieved. The production is not limited to cells, but can be extended to genetically engineered transgenic plants (Biemelt and Sonnewald 2004).

Against this backdrop, one expects this technology to be at the frontier of modern medical research and industry—but just the opposite is true. As proven by different comparative studies published by the EU, plants are—on a theoretical level, potentially and relative to e.g. human embryonic stem cells, animal models etc.—the most secure and most cost-effective alternative when the production of pharmaceutical-proteins is concerned (Spoek and Karner 2008; Goldstein and Thomas 2004; Ma et al. 2004). Nevertheless, this promising approach is not applied in a broader dimension, although there is no sufficient scientific or technological reason not to use the achievements attained by biotechnology. There is one leading reason for this paradox: The point when the green light turns to red is reached, when public perception and opinion—or in other words: societal concern and unease—are considered. This can be recently seen as the crucial point in the discussion—initiated by the European Commission—about a possible relaxation for the use of different products of the so called ‘green biotechnology’ in the field of nutrition (European Commission 2011).

In sum, biotechnological and life science innovations do not only lead to immense progress in diverse fields of natural science and technical research and thereby drive economic development; they also fundamentally affect the relationship between nature, technology and society. What can be done to frame this complex and perhaps chaotic field and to develop strategies for dealing with these challenges? In order to map this vague and stirring field, we felt forced to think about and develop a tool which firstly takes the reciprocal interaction at the interface of science and society (especially the media driven society) serious and secondly gives us the opportunity to detect possible junctions of unease before they appear. Therefore, we propose the concept of ‘bio-objects’. This term has been recently proposed and is in need of further clarification and conceptual work.

## ‘Bio-objects’ as a heuristic device to identify potential conflicts at the interface of science and society

Against the background of the sketched current trends of how science and society meet, ‘bio-objects’ are entities, which can be characterized by three constitutively interconnected features:

1. They have been isolated from their natural contexts (organs, individual cells and microorganisms, sub-cellular structures) and subjected to further procedures in order to be utilized in medical and life-science contexts (Vermeulen et al. 2012).
2. Additionally, as products of scientific and technological processes, ‘bio-objects’ share some characteristics with organic structures and thus they seem to belong to the realm of life. But as shown above, they differ fundamentally from other organic entities (‘things’) in the sense that they are also subjects of current—media alerted—public debates, which affirmatively and/or critically accompany the research process in the form of an ethical, legal, social, medial and political discourse.
3. A further attribute of ‘bio-objects’ is their potential for economization, which is not limited to the monetization or capitalization of ‘bio-objects’ themselves, but may also refer to the use of ‘bio-objects’ in value creation processes more generally, even if this value cannot yet be precisely quantified.

By using such a concept of ‘bio-objects’ we are able to systematically line out and examine the interdependency of these three dimensions concerning all ‘cutting edge biotechnologies’. In concrete terms: the so characterized ‘bio-objects’—not only products of synthetic biology, but also hES or iPS cells-waver between being a scientific and societal ‘benefit’ or ‘risk’.

Reformulating this point with Latour (1993), one of the world’s most important protagonist in the field of science and technology studies, ‘bio-objects’—though not explicitly entitling this concept—seem to take on a life on their own and resist to be subsumed under the category of ‘things’. In their media presence and their provocation of social discourse, products of synthetic biology, stem cells or animal models themselves start acting and thus escape the exclusive control and disposal of the actors who produce them. By developing such an ‘independent existence’, they *appear* as subjects—or with others words: actants (Latour 2007)—standing alongside classical actors, such as scientists, politicians, patients, etc., in partly cooperative and partly conflicting interactions.

Due to such a resistance of ‘bio-objects’ it is a current “matter of concern” (Latour 2008) that it is not yet clear if they will be perceived as societal risk or benefit. Nevertheless, the outcome will be decisive for the public perception and evaluation both of ‘bio-objects’ themselves and of associated research processes like synthetic biology. The intended and/or expected availability of ‘bio-objects’ for certain purposes—in the fields of medicine, energy generation and storage, ecology, agriculture, food production, etc.—will be constitutive for their public perception, for the flow of financial resources for research and for the attention of the media.

As shown above, it is all the more urgent to conduct a thorough and timely interdisciplinary exploration of ‘bio-objects’, in order to take up the described conceptual challenges. This holds true at least if science and politics wish to identify adequate, sufficiently complex responses and feed them into the public debate. Taken this seriously, it is also a crucial point to scrutinize the ‘evolution’ of the ‘bio-objects’ concerning the interaction between the past and the currently emerging ‘bio-objects’. Therefore, the modeling of this cluster of problems between the poles of ‘nature’, ‘technology’ and ‘society’ represents an urgent research goal, especially since a viable strategy for dealing with these challenges and questions *in advance* has yet to be developed.

**Fig. 1:**
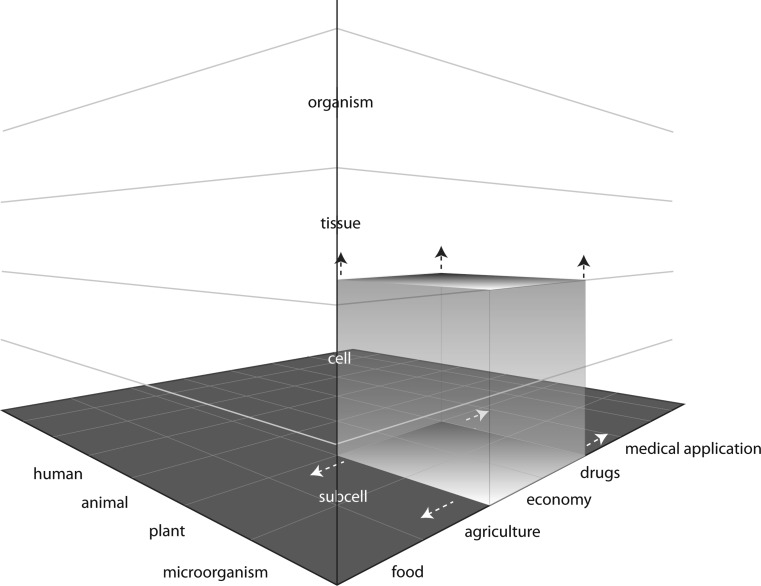
Model to survey the world of ‘bio-objects’. This model illustrates the multi-dimensionality of bio-objects. Hereto the different dimensions of domain (microorganism, plant, animal and human), the degree of complexity (sub-cellular, cellular, tissue, and organism) and finally the respective societal application context (food, agriculture, economy, drugs, medical application) are interlinked with each other. Newly appearing ‘bio-objects’ can be placed in this matrix (*grey cuboid*) and this model enables to observe and detect the transformation of such ‘bio-objects’ trough different dimensions

In order to identify, observe and meet these challenges, we propose a three-dimensional matrix that will provide orientation in the so far unexplored world of ‘bio-objects’ (Fig. 1).

The suggested model considers the multi-dimensionality of ‘bio-objects’ and enables the integration of newly appearing ‘bio-objects’, which is an obvious and essential goal posed by the dynamics in biotechnology as well as the exposed societal challenges. The products of plant-based synthetic biology, for example, can scientifically be limited to the application fields of economy and drugs up to now. But as the mentioned example demonstrates, ‘bio-objects’ start shifting through the various dimensions driven by the scientific community, the media or civil-society-organizations. The proposed model enables to uncover the transformation and the shift of ‘bio-objects’ through various dimensions. Finally, this methodological approach allows a retrospective as well as a prospective observation of existing and upcoming ‘bio-objects’ in science, technology and society. Such an *prospective* observation and understanding is a required necessity in order to deal with the rising societal discontent.

An exploration of the subsequent governance challenges, however, presupposes the exact determination of ‘bio-objects’ with regard to their respectively associated place within the matrix: Concerning synthetic biology it is still completely open along which of the three dimensions (domain, degree of complexity, societal application context) ‘bio-objects’ will be discussed primarily, how they are going to be received and evaluated by society and which effects they will have on the protagonists themselves. In relation to these urgent questions it is also still open wether synthetic biology will be associated with the currently neutral connoted ‘white biotechnology’ or the rather negative connoted ‘green biotechnology’. At this point, the developed matrix provides the possibility to precisely identify the different association possibilities of different actors (scientists, CSOs, media, etc.), the possible disruptions of previous distinctions as well as the combination of both acts as a driver for different measurement possibilities. Based on such an analysis of the possible path- and crossways of the emerging ‘bio-objects’ it is also possible to identify the potential links for a rising societal concern and unease. To be sure, this obviously doesn’t mean that it is possible to determine all crucial points but to calibrate the up- and downstreaming waging between science, technology and society more precisely (Nuffield Council on Bioethics 2012). All this has to be noticed *before* a new technology is brought into action. It is too late and (on a societal level as well as economically) much too dangerous to wait for the demand of a moratorium until the effectiveness and resistance of ‘bio-objects’ at the interface of science and society is taken seriously.

Therefore, the ethical and societal assessment of synthetic biology is challenged not only to constrain on questions of biosafety and biosecurity but also to face the questions in synthetic biology as an interface problem of science and society triggered by societal concerns and unease. Furthermore, an interdisciplinary discussion on ‘bio-objects’ is among the most urgent desiderata of scientific research in order to trace the emergence of ‘bio-objects’ and the questions and conflicts evoked by them in an adequate way. There will be no ‘exit’ if ‘white biotechnology’ is to be developed successfully.
